# Neuroendoscopic surgery for ventriculitis and hydrocephalus after shunt infection and malfunction: Preliminary report of a new strategy

**DOI:** 10.1111/ases.12162

**Published:** 2015-04-24

**Authors:** Sadaharu Tabuchi, Mitsutoshi Kadowaki

**Affiliations:** Department of Neurosurgery, Tottori Prefectural Central HospitalTottori, Japan

**Keywords:** Hydrocephalus, septostomy, ventriculitis

## Abstract

If not controlled in the early stage, ventriculitis is difficult to treat neurosurgically and can lead to serious sequelae, a long course of treatment, and hospitalization. We report two cases of ventriculitis and progressive hydrocephalus after shunt infection. Both were successfully treated by neuroendoscopic septostomy in combination with thorough intraventricular irrigation through a single burr hole followed by single shunt revision. Although surgical intervention has not been established as a first-choice treatment for ventriculitis, including early-stage ventriculitis, prompt neuroendoscopic surgery appears effective for the management of ventriculitis and hydrocephalus after shunt infection. The strategy described in this report might be useful to avoid recurrent shunt infections and malfunctions, simplify a shunt, and reduce the overall duration of hospitalization.

## Introduction

Bacterial ventriculitis is a difficult condition to treat in neurosurgical practice if it is not controlled in the early stage. It causes brain abscess, adhesion, and septation inside the ventricles, and can lead to a long course of treatment and hospitalization. Because ventriculitis is a severe intracranial infection that can lead to serious sequelae and death, prompt diagnosis and treatment are necessary. We show that early, aggressive neuroendoscopic treatment for ventriculitis and progressive hydrocephalus may be effective in reducing the rate of further shunt infection and/or malfunction and the duration of hospitalization.

## Case Presentation

### Case 1

**Figure 1:**
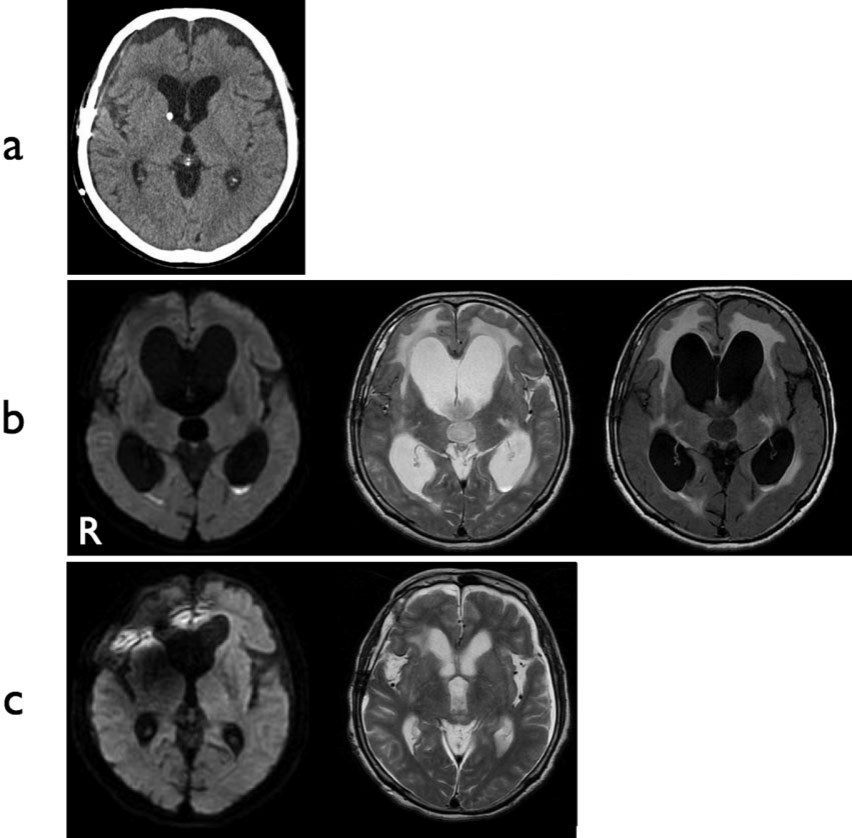
Case 1. (a) CT after the VP shunt had been placed and 2 months before this admission. (b) MRI on admission. Diffusion-weighted imaging (DWI) shows abnormal hyperintensities in the trigones of both lateral ventricles, suggesting debris (left panel). T_2_-weighted imaging (central panel) and fluid-attenuated inversion recovery imaging (right panel) show abnormal periventricular intensities. (c) Postoperative MRI. DWI shows disappearance of hyperintense lesion in the ventricles. Hydrocephalus has resolved. A metallic artifact from the shunt system is present in the right frontal region. VP, ventriculoperitoneal.

A 73-year-old woman suffering from decreased activity and high fever was admitted because her ventriculoperitoneal shunt, which had been inserted 4 months previously for normal-pressure hydrocephalus after subarachnoid hemorrhage, had malfunctioned and become infected. Before hospitalization, the patient's modified Rankin Scale score was 4 (Figure 1a). MRI showed evidence of ventriculitis, including intraventricular debris in the trigones of both lateral ventricles on diffusion-weighted imaging and abnormal periventricular intensities on fluid-attenuated inversion recovery imaging (Figure 1b). Examination of cerebrospinal fluid (CSF) showed an increased white blood cell count and decreased glucose level. CSF culture revealed *Corynebacterium striatum*. Although the shunt system was removed immediately and antibiotic treatment was initiated, the infection persisted despite intravenous administration of antibiotics including cefazolin 2 g/day, meropenem 6 g/day, and vancomycin 1.5 g/day for 3 weeks.

**Figure 2:**
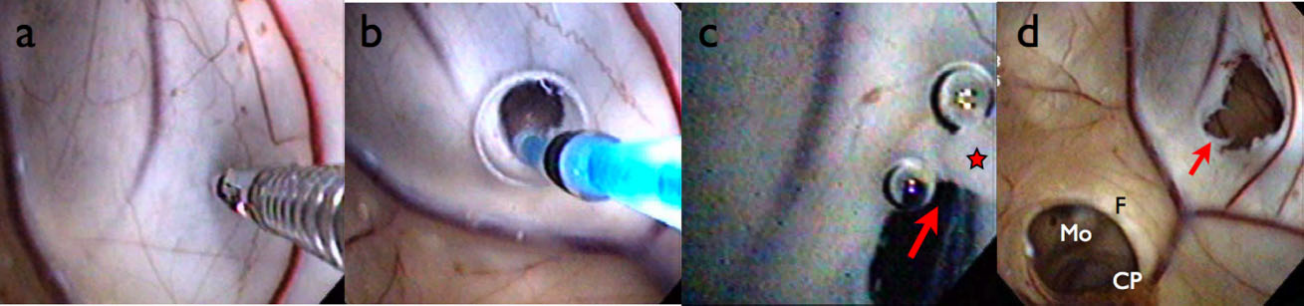
Case 1, intraoperative findings and surgical procedures. (a) Initially, perforation of the septum was performed using biopsy forceps. (b) The small hole was expanded using a 3-Fr balloon catheter. (c) The hole was further extended by monopolar cutting (arrow) to a diameter of at least 5 mm.. (d) The septal fenestration (arrow) was completed. Through this, the contralateral ventricle was clearly observed and accessible. CP, colloid plexus; F, fornix; Mo, foramen of Monro; star, monopolar probe.

Disturbance of consciousness occurred due to the progression of hydrocephalus, so intermittent lumbar puncture and removal of CSF was performed, but it failed to prevent the progression of hydrocephalus. To improve the status of the patient, extensive ventricular irrigation using commercially available artificial CSF (ARTCEREB; Otsuka Pharmaceutical Factory, Tokushima, Japan) was attempted under neuroendoscopy using a flexible videoscope (VEF-V; Olympus Medical Systems, Tokyo, Japan) with satisfactorily clear images and operability (1–3). A left frontal burr hole was created for the approach. Multiple dirty yellowish-orange microgranulations and abnormal substances were observed in the lateral and third ventricles. Septostomy was performed with biopsy forceps, a 3-Fr balloon catheter, and monopolar cutting to allow for effective and thorough irrigation of both lateral ventricles through a single burr hole (Figure 2a–d). The videoscope was then inserted into the contralateral ventricle through this opening and irrigation was performed. Floating materials were sufficiently washed out by neuroendoscopic irrigation. The videoscope was also inserted into the third ventricle through the foramen of Monro, and sufficient irrigation was achieved. This surgical procedure resulted in resolution of the infection within 2 weeks postoperatively. After it was confirmed that CSF showed normal characteristics, single ventriculoperitoneal shunt revision was performed successfully, and the patient's level of consciousness rapidly improved (Figure 1c). No shunt infection or malfunction has occurred as of 14 months postoperatively. The patient is now able to walk with mobility aids, eat meals by herself, and live independently at home.

### Case 2

**Figure 3:**
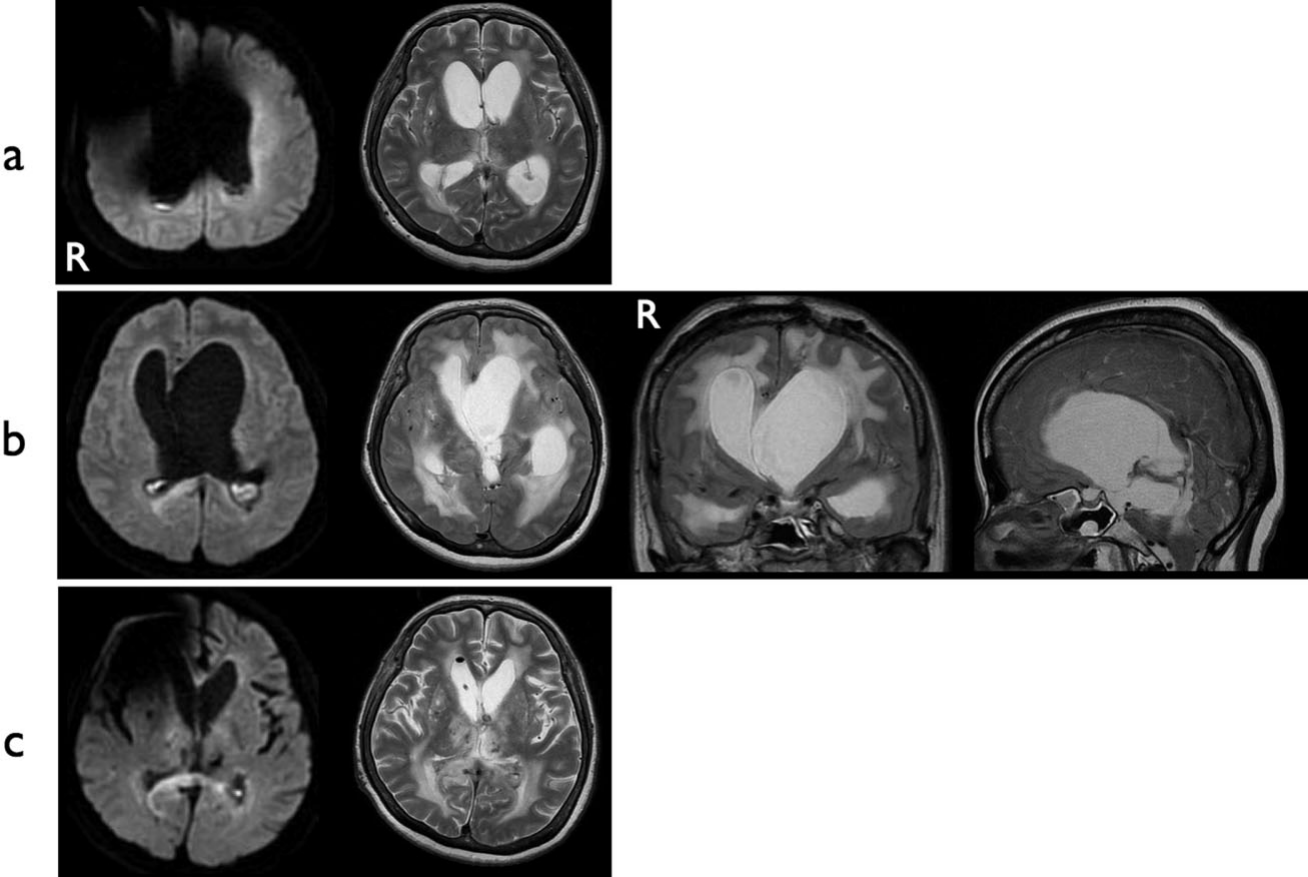
Case 2. (a) MRI on admission. DWI shows abnormal hyperintensities in the occipital horns of both lateral ventricles. Hydrocephalus is obvious. A metallic artifact is apparent around the right frontal region. (b) Preoperative MRI. Progression of asymmetrical hydrocephalus with findings of ventriculitis is evident. (c) Postoperative MRI. Both ventriculitis and hydrocephalus have resolved. DWI, diffusion-weighted imaging.

A 56-year-old woman was admitted a second time because her ventriculoperitoneal shunt, which had been inserted 8 months previously for normal-pressure hydrocephalus after thalamic hemorrhage with intraventricular hematoma, had malfunctioned and become infected. Before hospitalization, the patient's modified Rankin Scale score was 5. Examination of the CSF showed an increased white blood cell count and a dramatic increase in protein level (807 mg/dL). CSF culture yielded negative results. MRI showed findings consistent with ventriculitis (Figure 3a).

The shunt system was immediately removed, and antibiotic treatment was started. The infection gradually improved over 4 weeks with aggressive intravenous antibiotic treatment using ceftriaxone 2 g/day, vancomycin 2 g/day, and panipenem 4 g/day. However, asymmetrical hydrocephalus developed during treatment, and progressive expansion of the ventricles was marked (Figure 3b). Neurological findings progressively deteriorated, with the appearance of anisocoria and disappearance of both pupillary light reflexes and eyelash reflexes. This showed obstructive hydrocephalus secondary to ventriculitis, which necessitated urgent surgical treatment.

**Figure 4:**
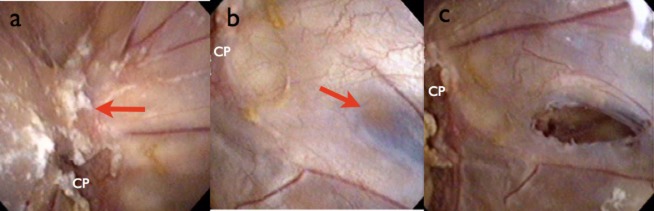
Case 2, intraoperative findings. (a) The left foramen of Monro was firmly obstructed by inflammatory substances that represented an obstacle for foraminoplasty (arrow). (b) A thin, semi-transparent portion (arrow) of septum was identified posterior to the anterior septal vein. Fenestration was performed in this portion using the techniques mentioned in Figure 2. (c) The septal fenestration was successfully completed. CP, colloid plexus.

Under neuroendoscopic observation through the left frontal burr hole, the foramen of Monro was seen to be obstructed by inflammatory reaction (Figure 4a), leading to asymmetrical ventricular enlargement. To restore CSF circulation within both lateral ventricles, neuroendoscopic septostomy was performed (Figure 4b,c). The endoscope approached the right lateral ventricle through this septal fenestration and the third ventricle through the right foramen of Monro. Neuroendoscopic irrigation of the ventricles was effectively performed. After surgery, CSF results improved over the course of a week, and single-shunt revision was performed successfully. Follow-up MRI showed that shifting of the septum pellucidum had resolved and hydrocephalus was dramatically improved (Figure 3c). No further shunt infection or malfunction has been seen as of 1 year postoperatively. The patient is now receiving care in a nursing home.

Both cases showed rapid improvement of ventriculitis and hydrocephalus after implementing these procedures.

## Discussion

Bacterial ventriculitis, which can lead to purulent brain abscess, remains an important cause of mortality and morbidity in patients with hydrocephalus. Rapid drainage and irrigation of pus and debris may be beneficial for reducing inflammation and preventing intraventricular septation and subsequent isolated ventricular enlargement. Early diagnosis of ventriculitis may significantly improve the overall outcome, and MRI plays an important role as a first-line diagnostic tool (4). The most frequent signs of ventriculitis have been reported to be intraventricular debris and pus. Diffusion-weighted imaging is a highly sensitive modality for detecting ventriculitis (4). Laboratory data for CSF (positive cultures or increased white cell counts and protein levels), clinical information, and MRI features (ventricular debris, periventricular hyperintensity) are sufficient to diagnose ventriculitis (5), and findings for both of our patients were consistent with this diagnosis.

In the present cases, as hydrocephalus progressed and neurological status worsened during the course of treatment, both patients required prompt CSF diversion, specifically shunt surgeries. However, such shunts are likely to become infected more frequently than routine shunts in naive or non-infected cases, and they also carry a risk of malfunction due to obstruction by debris or proteinaceous materials.

Even after a shunt has been removed, ventriculitis may persist despite intravenous and/or intrathecal administration of antibiotics. In such cases, further treatment options must be considered. Continuous intraventricular irrigation therapy using a closed drainage system has been reported (6–9). Intraventricular and lumbar intrathecal administration of antibiotics in cases of serious ventriculitis has been reported to lead to quick CSF stabilization (10). However, excessive and long-term external drainage of CSF must be avoided because of the risk of aggravating bacterial ventriculitis by retrograde infection (11).

Schulz *et al*. recently reported the benefits of endoscopic irrigation for ventriculitis as an adjunct to continuous antibiotic treatment in newborn infants (12). Although surgical intervention has not been established as a treatment of first choice for ventriculitis (13), including early-stage ventriculitis, we consider prompt neuroendoscopic surgery to be effective for the management of ventriculitis and hydrocephalus after shunt infection.

Septostomy is a safe and efficient technique to restore CSF circulation in cases of unilateral hydrocephalus (14,15). Septostomy is also a useful technique for effective intraventricular irrigation through a single burr hole, as described in this study. We propose septostomy combined with thorough intraventricular irrigation using artificial CSF as a useful technique for ventriculitis and hydrocephalus. The strategy described in this report might be useful to avoid recurrent shunt infections and malfunctions, or to simplify a shunt. This strategy may help to reduce the overall treatment and hospitalization periods and to improve outcomes.

We prefer using a flexible endoscope rather than a rigid endoscope in these kinds of cases because of its operability and the ability to perform septostomy and ventricular irrigation through a single burr hole (2,3,15,16).

The procedures used in this study show some similarities to endoscopic surgery using a flexible endoscope for intraventricular hemorrhage; however, the access is bilateral in cases with bilateral hematoma (16), and our method always approaches from only one side, necessitating septostomy in every case. Although previous reports have described the use of Ringer's solution for endoscopic ventricular irrigation (16,17), we have used a commercially available artificial CSF since 2009, as this product is physiologically superior to Ringer's solution for the brain (1).

According to our experiences and a previous report (12), we believe that the appropriate timing for neuroendoscopic surgery may be around 2 weeks after aggressive intravenous antibiotic treatment and confirmation of unsatisfactory effect after the diagnosis. The optimal timing of surgery should be examined in a further study.

A key drawback of this study was the small number of cases. A larger series or case-control study is needed to elucidate the effectiveness of this strategy.
